# Optimization and Biodistribution of [^11^C]-TKF, An Analog of Tau Protein Imaging Agent [^18^F]-THK523

**DOI:** 10.3390/molecules21081019

**Published:** 2016-08-05

**Authors:** Yanyan Kong, Yihui Guan, Fengchun Hua, Zhengwei Zhang, Xiuhong Lu, Tengfang Zhu, Bizeng Zhao, Jianhua Zhu, Cong Li, Jian Chen

**Affiliations:** 1 PET Center, Huashan Hospital, Fudan University, Shanghai 200235, China; karenyanyan@hotmail.com (Y.K.); fengchun_hua@163.com (F.H.); pet_111@126.com (Z.Z.); xiuhong_lu2015@sina.com (X.L.); 2 Department of Pathology, Shanghai Medical College, Fudan University, Shanghai 200030, China; tengfangzhufdu@gmail.com; 3 No.6 Shanghai People’s Hospital, Jiaotong University, Shanghai 200235, China; bizeng_zhao@sina.com; 4 Key Laboratory of Smart Drug Delivery, Ministry of Education & PLA, School of Pharmacy, Fudan University, Shanghai 201203, China; zhujianhua_fudan@126.com (J.Z.); congliphar@126.com (C.L.); chenjian_david@yeah.net (J.C.)

**Keywords:** Alzheimer’s disease, positron emission tomography (PET), tau, neurofibrillary tangles (NFTs), imaging

## Abstract

The quantification of neurofibrillary tangles (NFTs) using specific PET tracers can facilitate the diagnosis of Alzheimer’s disease (AD) and allow monitoring of disease progression and treatment efficacy. [^18^F]-THK523 has shown high affinity and selectivity for tau pathology. However, its high retention in white matter, which makes simple visual inspection difficult, may limit its use in research or clinical settings. In this paper, we optimized the automated radiosynthesis of [^11^C]-TKF and evaluated its biodistribution and toxicity in C57 mice. [^11^C]-TKF can be made by reaction precursor with [^11^C]MeOTf or ^11^CH_3_I, but [^11^C]MeOTf will give us higher labeling yields and specific activity. [^11^C]-TKF presented better brain uptake in normal mouse than [^18^F]-THK523 (3.23% ± 1.25% ID·g^−1^ vs. 2.62% ± 0.39% ID·g^−1^ at 2 min post-injection). The acute toxicity studies of [^11^C]-TKF were unremarkable.

## 1. Introduction

Aging and declining mental health in later life is a principal socioeconomic challenge of the 21st century. The World Health Organization estimated that nearly 36 million people were affected by Alzheimer’s disease (AD) in 2012, and this number is expected to double by 2030, and more than triple by 2050. Given the scale of the problem, new tools for understanding and eventually treating AD are urgently required [1].

Tau proteins, which are associated with the stabilization of microtubules, may be abnormally phosphorylated and form paired helical filaments (PHFs) in AD patients’ brains. PHFs finally assemble into neurofibrillary tangles (NFTs) and neuropil threads, causing dystrophic neuritis [2,3,4]. Neurofibrillary lesions appear in certain brain areas before the onset of dementia, and autopsy studies indicate a higher level of correlation between tau pathology levels and cognitive dysfunction when compared to Aβ pathology, indicating the presence of NFTs in the brain is a hallmark feature of AD [5,6]. Therefore, quantitative imaging of the tau burden may offer the opportunity for in vivo topographical mapping and quantification of tau aggregates in parallel with clinical and cognitive assessments. It is also helpful in evaluating the therapeutic effect of longitudinal tau-targeted AD treatments. Exploration of the living human brain in real time and in a non-invasive way was only theoretical for centuries; however, it has become possible today with the remarkable development of powerful molecular imaging techniques, especially positron emission tomography (PET), which was developed during the last four decades. Molecular PET imaging relies, from a chemical point of view, on the use and preparation of a positron-emitting radiolabeled probe or radiotracer. In this regard, non-invasive imaging with radiotracers for PET serves as a unique tool for quantifying spatial and temporal changes in characteristic biological markers of brain disease and for assessing potential drug efficacy. The present study focused on the development of novel NFT-targeting PET imaging agents for the investigation of AD pathogenesis.

Recently, several PET radiopharmaceuticals targeting abnormal conformations of the tau protein have been developed. Tau imaging is considered of significant importance for earlier and more accurate diagnosis of tauopathies, monitoring of therapeutic interventions and drug development. Here, we shed light on the most important developments in tau radiopharmaceuticals and highlight challenges, possibilities and future directions. Harada R et al. and Shcherbinin S et al. demonstrated the in vivo binding ability of THK5351 and AV1451 in patients with Alzheimer’s disease [7,8]. Other tau imaging agents, i.e., [^18^F]-THK523, [^18^F]-THK5105, [^18^F]-THK5117, [^18^F]-T807, [^18^F]-T808 and [^11^C]-PBB3, have been described and are considered promising as potential tau radioligands [9]. Kolb and colleagues have reported ^18^F-labeled tau tracers, i.e., [^18^F]-T807 and [^18^F]-T808. These tracers showed promising results in both in in vitro and in vivo studies [10,11,12]. [^18^F]-THK-5105 and [^18^F]-THK-5117 have more preferable properties as PET tau imaging radiotracers compared with [^18^F]-THK523 [13]. Tago reported that the 2-arylhydroxyquinoline derivative [^11^C]-THK951 demonstrated excellent kinetics in a normal mouse brain (3.23% ID/g at 2 min post-injection and 0.15% ID/g at 30 min post-injection) and showed the labeling of NFTs in an AD brain section by autoradiography assay, indicating the availability of [^11^C]-THK951 for in vivo PET imaging of tau pathology in AD [14,15]. Previous studies demonstrated that [^18^F]-THK523 had high affinity and selectivity for tau pathology both in vitro and in vivo [16]. Comparing the binding properties of [^18^F]-THK523 and other amyloid imaging agents including [^11^C]-PiB, [^11^C]-BF227 and [^18^F]-FDDNP, [^18^F]-THK523 showed higher affinity than other probes for tau fibrils [17,18]. [^18^F]-THK523 selectively binds to paired helical filament tau in AD brains but does not bind to tau lesions in non-AD tauopathies, such as corticobasal degeneration (CBD), progressive supranuclear palsy (PSP) and Pick’s disease (PiD), or to α-synuclein containing Lewy bodies in PD brains [19]. However, its high retention in white matter makes simple visual inspection of the images difficult, limiting its use in research or clinical settings [18]. Given the fact that carbon-11 radiolabeling would not change the pharmacokinetics and pharmacodynamics of the target compound, we radiosynthesized [^11^C]-TKF as PET tracers on the basis of [^18^F]-THK523 for tau pathology monitoring and studied its in vivo biodistribution and acute toxicity in C57 mice.

## 2. Materials and Methods

The precursor of [^11^C]-TKF, 4-(6-(2-fluoroethoxy)quinolin-2-yl)aniline (THKF-2), was synthesized by our research group [20,21]. Triflate-Ag was purchased from Sigma-Aldrich Corporation (St. Louis, MO, USA). Acetonitrile and ethanol of HPLC grade were obtained from Shanghai Lingfeng Chemical Reagent Co., Ltd. (Shanghai, China). Sep-Pak tC18 solid phase extraction (SPE) cartridge (78.4 μm of particle size) and sterile filters (0.22 μm) were purchased from Waters Corporation (Milford, MA, USA).

The [^11^C]-TKF automated synthesis module (TRACERlab FX_c_) was purchased from GE medical system. Semi-preparative high-performance liquid chromatography was conducted using a Waters pump (Waters Corporation) with a Bioscan radioactivity detector. Analytical radio-HPLC (Waters Corporation) was equipped with a dual λ absorbance detector (Waters 2487), binary HPLC pump (Waters 2487) and a Bioscan radioactivity detector. NMR and LC-MS were purchased from Bruker Corporation (Karlsruhe, Germany).

### 2.1. Chemistry

#### Preparation of Reference Standard CTKF

THKF-2 (1.5g, 5.32 mmol) was dissolved in 2 mL dimethylsulfoxide (DMSO). KOH (1 g, 17.86 mmol) and CH_3_I (0.745 g, 5.25 mmol) were then added. The solution was heated to 125 °C and stirred for 5 min. A mixture of CH_3_OH/HCI (*v*/*v* = 2/1) in 4.5 mL was added to the above solution and then stirred for 5 min at the same temperature. All the above reactions were carried out under nitrogen. The solution was poured into ice water (50 mL) and adjusted to pH 7.0 with sodium acetate (2 mmol). The resulting reaction mixture was loaded onto a Sep-Pak C-18 column and followed by washing with 10 mL of H_2_O and rapid air bolus. The final product CTKF was eluted by 2 mL of ethanol. Evaporation of the solvent gave a white solid, which was recrystallized in diethyl ether and *n*-hexane to give 4-(6-(2-fluoroethoxy)quinolin-2-yl)-*N*-methylaniline (CTKF, 1.32 g, 4.468 mmol) in 84% yield. 8.20–8.06 (m, 4H, Ar), 7.78 (d, *J* = 8.7 Hz, 1H), 7.39 (dd, *J* = 9.2 Hz, 2.8 Hz), 6.71–6.76 (m, 2H, Ar), 4.75–4.94 (dm, CH_2_F), 4.29–4.41 (dm, CH_2_O), 3.95 (br, NH), 2.92(d, *J* = 2.8 Hz, CH_3_).

### 2.2. Radiochemistry

#### 2.2.1. Radiosynthesis of [^11^C]-TKF Using [^11^C]MeOTf

The synthesis of [^11^C]-TKF was fully automated using a TRACERlab FX_c_ automated system. High specific radioactivity [^11^C] methyl iodide was synthesized from [^11^C] carbon dioxide which was produced by Eclipse HP cyclotron (15 min bombardment). ^11^CO_2_ was trapped onto an oven packed with molecular sieve and Ni-catalyst, then filled with hydrogen and heated at 400 °C to make ^11^CH_4_. Methane was transferred to quarts oven mixed with iodine gas at 720 °C. [^11^C] methyl iodide (5600 MBq) was trapped onto Porapak N trapper, recirculated for 6 min. Then Porapak N trapper was heated to 190 °C, the released [^11^C] methyl iodide path through an Ag-Triflate column which was heated at 190 °C by a stream of helium gas (30 mL/min). [^11^C] methyl iodide was converted to [^11^C]MeOTf, and trapped into the reaction vessel containing 0.7–1 mg THKF-2 in 0.5 mL dry acetone. After the [^11^C]MeOTf was trapped in the reaction vial, the mixture was heated at 80 °C for 3 min. Trapping was monitored by GM detector until the maximal value was attained. The resulting mixture was subjected to a prepurification procedure using (solid-phase extraction) prior to semipreparative HPLC purification. Acetone was evaporated with a flow of nitrogen gas and the residue was dissolved in 0.5 mL of acetonitrile. The resulting reaction mixture was loaded onto semi-preparation HPLC (EtOH/H_2_O = 60/40 (*v*/*v*), flow rate 3 mL/min). The desired fraction was collected and diluted with 100 mL of distilled water, then passed through a SepPak^®^ C18 light cartridge that was pre-conditioned with ethanol (8 mL) and water (12 mL). The product was trapped on the C18 cartridge, and eluted from it with 1 mL ethanol to avail which contains 9 mL 0.9% sodium chloride. 5 mL 10% ethanol in saline which contain 2 mg (0.011 mmol) ascorbic acid as a stabilizer was added before sterile filtration through a 0.22 µm membrane filter into a sterile vial. Radiochemical purities and identity were determined by the co-injection with the reference standard CTKF in radioactive HPLC chromatogram.

#### 2.2.2. Radiosynthesis of [^11^C]-TKF Using ^11^CH_3_I

**Scheme 1:**
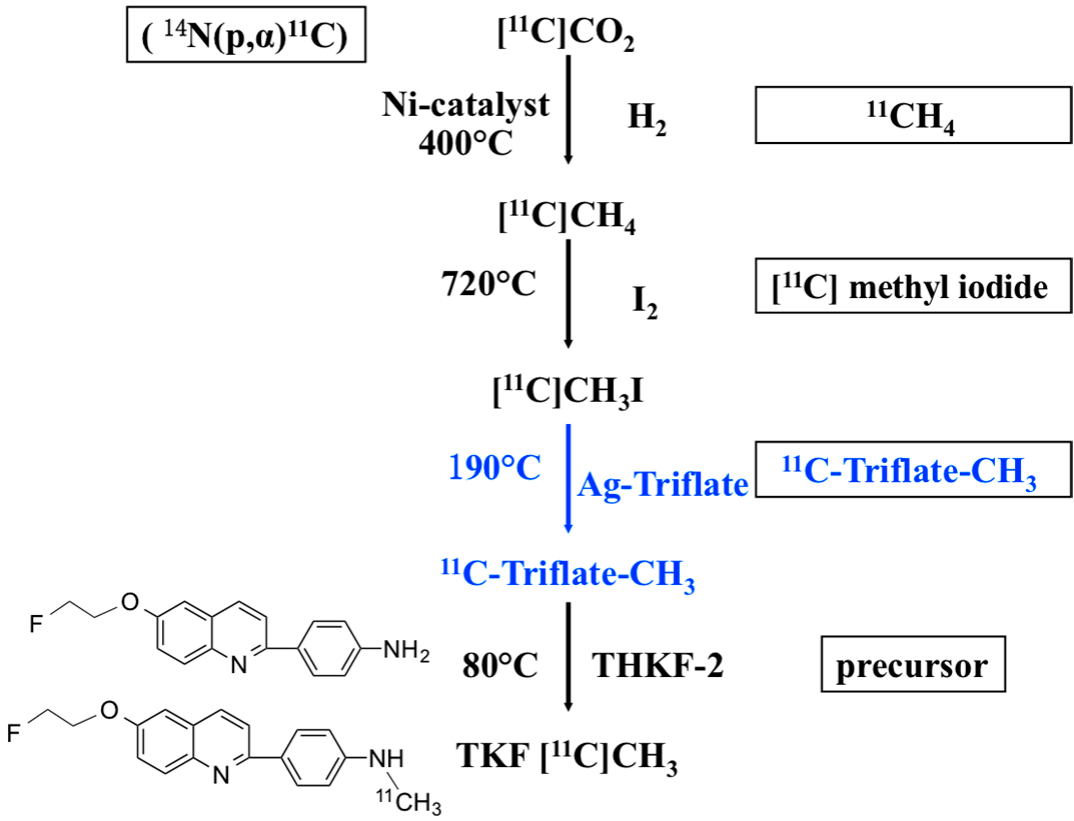
Radiosynthesis of [^11^C]-TKF (radiosynthesis of [^11^C]-TKF using [^11^C]MeOTf, and ^11^CH_3_I).

The difference of radiolabeling procedure for [^11^C]-TKF between using ^11^CH_3_I and [^11^C]MeOTf is whether to produce the intermediate [^11^C]MeOTf or not (Scheme 1).

### 2.3. Quality Control

Radiochemical purity and specific activity were evaluated by analytical HPLC. C18 reversed phase column (Purospher^®^ STAR LPRP-18e endcapped ((5 μm), 250 mm × 4.6 mm, mobile phase: Acetonitrile/water (75/25), flow rate: 1 mL/min UV at 350 nm). The retention time is 5.5 min [^11^C]-TKF.

### 2.4. Micro PET Imaging and Biodistribution Studies of [^11^C]-TKF

Normal C57 mice (20 ± 3 g) images were acquired with a Siemens Inveon PET/CT system (Siemens Medical Solutions, Knoxville, TN, USA). At the beginning of the PET scanning procedure, a CT scan (Inveon, Siemens Medical Solutions, Knoxville, TN, USA) was performed for all animals. [^18^F]-THK523 was given via the catheter system intravenously in a slow bolus. The total applied volume was 0.15 ± 0.05 mL. The amount of injected activity was 0.45 ± 0.05 mCi. Radioactivity in the syringe and catheter was measured immediately before and after injection. Dynamic data acquisition was performed by Inveon Acquisition Workplace (IAW, Siemens) for 60 min after injection (p.i.) of the tracer. The emission data were normalized and corrected for decay and dead time. The operation procedure, PET image reconstruction and analysis is carried out according to micro PET imaging and biodistribution studies of [^18^F]-THK 523 [19]. Time-sequential scanning was performed for 60 min in the three-dimensional (3D) list mode with an energy window of 350–650 keV. List-mode data were sorted into 3D sonograms as 21 frames (3, 20, 2, 30, 3, 60, 4, 150, 9, 300), followed by Fourier rebinning into two-dimensional sinograms. Dynamic images were reconstructed with filtered back- projection using a ramp filter. Regions of interest (ROI) were masked on the brain using PMOD software (version 3.403, PMOD technologies, Zürich, Switzerland). Decay-corrected radioactivity was expressed as SUV ((tissue radioactivity/milliliter of tissue)/(injected dose/gram of body weight)). ROI were masked on the brain, heart, lung, liver, gallbladder, kidney, stomach, small intestine, muscle, femur and blood using PMOD software. ROI in the blood were masked on the ventricular cavity using the frame of the first 20 s after administration. The areas under the curves (AUCs) of ROI in the tissue (AUCtissue, SUV*min) were calculated starting from 0 to 60 min.

The experiments were carried out in compliance with national laws for the conduct of animal experimentation and were approved by the local committee for animal research.

Results are presented as mean ± standard deviation.

### 2.5. Acute Toxicity Studies of CTKF

Toxicity studies of CTKF were performed at PET Center, Huashan Hospital and Department of Pathology, Shanghai Medical College, Fudan University. Acute toxicity was assayed in C57 mice. CTKF at a dose of 4.5 mg/kg body weight (0.45 mg/mL in physiological saline containing 0.01% (*w*/*v*) polysorbate 80) was injected intraperitoneally into four-week-old mice weighing 15–20 g and 14–19 g, for males (*n* = 5) and females (*n* = 5), respectively. The dose of 4.5 mg/kg body weight represents 70, 300-fold equivalent of the postulated administration dose (0.064 μg/kg body weight) of 500 MBq [^11^C]-TKF with a specific activity of 207 GBq/μmol for humans weighing 60 kg. The three lots of [^11^C]-TKF were also assayed after decay. [^11^C]-TKF was injected intravenously into four-week-old male and female mice (*n* = 5 for each) at doses of 2.2 μg/7.5 mL/kg body weight and 1.9 μg/7.0 mL/kg body weight, respectively, which was equivalent to 400-fold postulated administration dose of 500 MBq [^11^C]-TKF for humans. One lot (1.6 μg/6.8 mL/kg body weight), which was equivalent to 500-fold postulated administration dose, was also injected into four-week-old male and female mice (*n* = 5 for each). Animals were observed four times (0.5, 1, 3 and 6 h after the injection) on day 1 and thereafter once daily for clinical signs until 15 days, and weighed on days 4, 8 and 15. At the end of the 15-day observation period, the mice were euthanized and macroscopic analysis was performed. The control group was treated with the same volume of 0.9% saline. All dosing formulations were confirmed to be within ±10% of the target concentration for all groups. All dose levels were scaled to surface area for comparison with proposed human dosages. All animals were individually identified by ear punch and were observed at least once daily for signs of mortality, morbidity, injury, and availability of food and water. All observations were recorded daily (2–4 h post dose on the days of dose administration). Individual body weights were measured and recorded for each animal prior to dosing and at necropsy.

Histopathology was performed on both experimental and control groups. Sections of the tissues from animals to be evaluated were embedded in paraffin (5 microns thick) and stained with hematoxylin and eosin by Department of Pathology, Shanghai Medical College, Fudan University. Each lesion was listed and coded by the most specific topographic and morphologic diagnoses, as well as severity and distribution.

## 3. Results and Discussion

### 3.1. Radiochemistry

The reason for the special interest in carbon-11 is not only that carbon is present in virtually all biomolecules and drugs, but also that isotopic labeling of chemical structures of interest with this short-lived positron-emitting carbon-isotope will give radiotracers unchanged pharmacokinetics and pharmacodynamics when compared with the parent compound; in addition, a given molecule could be radiolabeled at different functional groups or sites, permitting us to explore (or to take advantage of) in vivo metabolic pathways. Carbon-11-methylation is by far the most frequently used method for the introduction of carbon-11 into organic molecules via the radiolabeled reagents [^11^C]methyl iodide ([^11^C]CH_3_I) and [^11^C]MeOTf ([^11^C]CH_3_O(SO_2_) CF_3_). Alkylation with ^11^CH_3_I or [^11^C]MeOTf is the most widely used method for introducing carbon-11 into target molecules. Various compounds have been prepared via N-, O- and S-methylation reactions. There are two common ways to prepare ^11^CH_3_I. In the “wet” method, ^11^CO_2_ is reduced to ^11^C-methanol by LiAlH_4_, followed by treatment with HI. In the “gas phase” method, ^11^CH_3_I is directly prepared from ^11^C-methane in the presence of iodine vapor. The natural abundance of CO_2_ in air is 330 ppm, whereas that of methane is 1.6 ppm. Therefore, precautions should be taken to exclude air from synthesis modules and solutions in order to get high specific activities. The use of [^11^C]MeOTf in methylation reactions has several advantages over the use of ^11^CH_3_I. Because [^11^C]MeOTf is far more reactive than ^11^CH_3_I, methylations can be conducted at lower reaction temperatures, with smaller amounts of precursor and shorter reaction times. The synthesis of [^11^C]MeOTf can be easily conducted as an on-line process by passing ^11^CH_3_I/^11^CH_3_Br through a column containing silver triflate that was pre-heated at 200–300 °C. The column containing the silver triflate needs to be stored in the dark and the column material should be free from oxygen.

[^11^C] methyl iodide and [^11^C]MeOTf were used in the majority of ^11^C preparations. A major reason is that a methylation reaction is simple and yields many biologically interesting radiopharmaceuticals. Initially, [^11^C]methyl iodide was prepared from ^11^CO_2_ that was trapped in a solution of LiAlH_4_ followed by the addition of HI. Because of the issue of specific activity, many PET centers prepare [^11^C]methyl iodide from ^11^CH_4_ in the gas phase using iodine vapor.

The decay-corrected radiochemical yield of the product [^11^C]-TKF obtained using [^11^C]MeOTf was >60% based on the activity of the [^11^C]methyl iodide trapped. The radiochemical purity of [^11^C]-TKF was >95%. The total synthesis time was 40 min including purification (from the end of bombardment).

By contrast, the yield of [^11^C]-TKF obtained by using ^11^CH_3_I was only >5%, and the radiochemical purity was >95%. The total synthesis time was 35 min including purification (from the end of bombardment).

**Figure 1:**
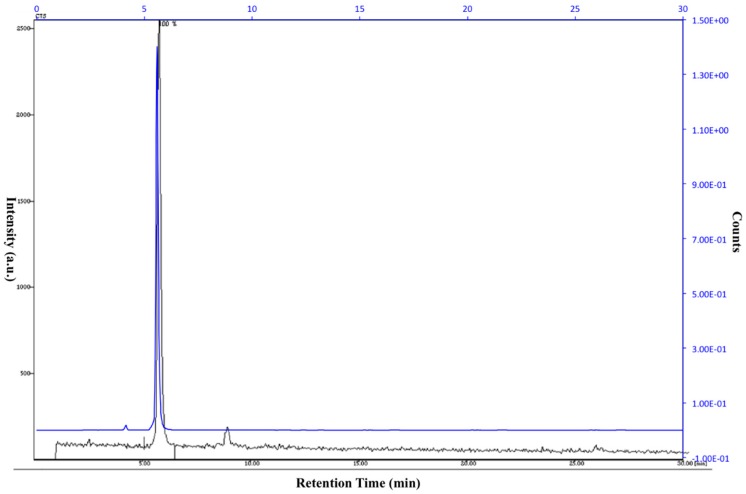
HPLC analysis of coinjection of CTKF and [^11^C]-TKF labeling. (Blue: UV detection, Dark: Radioactive detection: the retention time of CTKF and purified [^11^C]-TKF was about 5.5 min).

Thus, [^11^C]-TKF was labeled using [^11^C]MeOTf instead of [^11^C]methyl iodide. The use of [^11^C]MeOTf allowed milder reaction conditions, increased the radiochemical yield, decreased the amount of precursor required and reduced the total synthesis time of the procedure. The advantages of this new labeling method are summarized in Table 1. The [^11^C]MeOTf method induced a substantial improvement of radiosynthesis of [^11^C]-TKF. The radiosynthesis of [^11^C]-TKF was performed via [^11^C]MeOTf with high radiochemical yield (60%–65%), high radiochemical purity (>95%) and high specific activity (5.6 ± 0.3 Ci/µmol). The radiochemical purity and identity were determined by the co-injection with [^11^C]-TKF and the reference standard CTKF in a radioactive HPLC chromatogram. The same retention time peaks in the UV and the radioactive chromatograms were shown through the co-injection of [^11^C]-TKF and the reference standard CTKF. The retention time of the product [^11^C]-TKF on the analytical HPLC was approximately 5.5 min and the synthesis was completed in 40 min including purification (from the end of bombardment) in Figure 1.

### 3.2. Micro PET Imaging and Biodistribution Studies of [^11^C]-TKF

**Figure 2:**
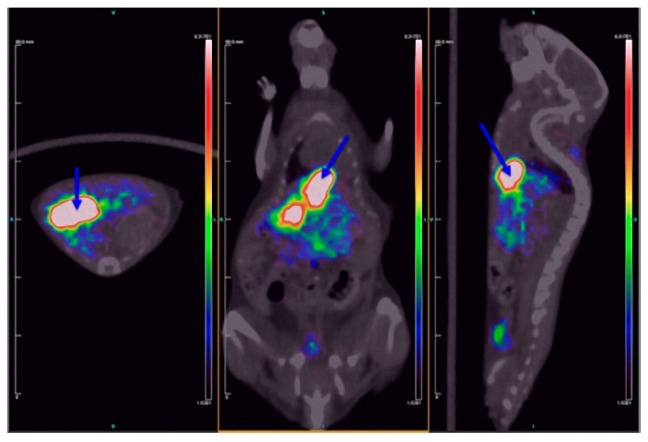
Micro PET of [^11^C]-TKF (Blue arrows indicate gall bladder: [^11^C]-TKF was mainly metabolized by the gallbladder and excreted through the biliary system).

The PET imaging showed a clear in vivo distribution of [^11^C]-TKF in C57 mice. [^11^C]-TKF was mainly metabolized by the gallbladder and excreted through the biliary system, thus leading to substantial rises in uptakes in the gallbladder and the intestines from 20 s to 60 min after injection, which were 6.18% ± 0.83% and 26.55% ± 3.70%, 6.16% ± 1.03% and 21.52% ± 3.54% ID·g^−1^, respectively (Figure 2). The uptake in the liver was the highest initially at 20 s (10.75% ± 0.93% ID·g^−1^), then it decreased with fluctuation. Additionally, the brain uptake was 3.23% ± 1.25% ID·g^−1^ at 2 min post-injection, which was higher than that of [^18^F]-THK523 (2.62% ± 0.39% ID·g^−1^ at 2 min post-injection) [19] (Table 2).

### 3.3. Acute Toxicity Studies of CTKF

**Figure 3:**
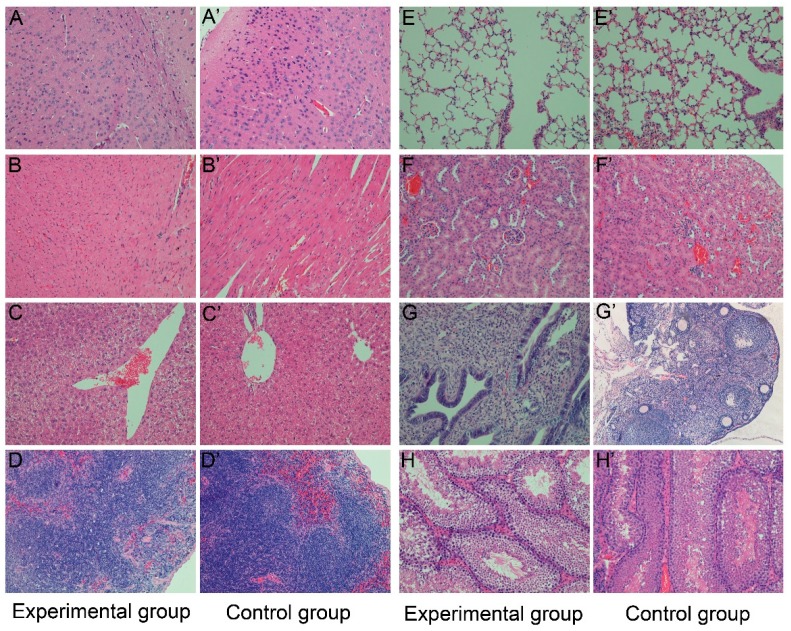
Pathological analysis of [^11^C]-TKF in C57 mice ex vivo. ((**A**,**A’**) No bleeding, edema, congestion, and inflammatory cell infiltration were observed in the brain tissues and meningeal tissues between experimental and control group; (**B**,**B’**) No abnormal myocardial necrosis and inflammatory cell infiltration were found in the heart tissues; (**C**,**C’**) Liver cells and periportal structure were intact without degeneration, necrosis or inflammatory cell infiltration; (**D**,**D’**) There were no congestion, capsule thickening or abnormalities in red and white medulla between experimental and control spleen; (**E**,**E’**) No congestion, dilation or inflammatory cell infiltration were found in alveolar capillary; (**F**,**F’**) No degeneration and inflammatory cell infiltration were observed in glomerular and tubular epithelial cell; (**G**,**G’**) No bruising, bleeding, or cyst formation were found in the ovary. Follicular development, the structure of endometrium, myometrium and outer membrane were normal. No hyperplasia and inflammatory cell infiltration were observed; (**H**,**H’**) No abnormalities were observed in the testicular structure).

Acute toxicity was evaluated after a single intraperitoneal administration of CTKF at a dose of 4.5 mg/kg body and a single intravenous administration of three lots of [^11^C]-TKF preparations in a dose range of 1.6–2.2 μg/kg. There was no significant difference between the experimental and control groups. No mortality was found in the mice. All of the rat groups showed normal gains in body weight compared with the control group, and no clinical signs were observed over a 15-day period. All animals survived until their scheduled sacrifice. No test article-related changes in body weights and food consumption were observed in the treatment groups compared with the control group. All tissues (brain, heart, liver, spleen, lung, kidney, ovary, uterus and testis) were examined histopathologically. No abnormalities were found on postmortem macroscopic examination (Figure 3).

## 4. Conclusions

Positron emission tomography works as a valuable tool for imaging the ongoing NFT processes in the central nervous system of AD patients. A new probe targeting the tau protein, [^11^C]-TKF, was designed for preliminary research. In addition, we tested different radiolabeling methods and established the automated radiosynthesis processes. The biological characteristics of [^11^C]-TKF were evaluated. Standard reference compounds of [^11^C]-TKF and CTKF were synthesized and identified. Radiosynthesis of [^11^C]-TKF via ^11^C-CH_3_I on an automated synthesis module was compared with that via [^11^C]MeOTf. In summary, the labeling yields and specific activity of [^11^C]-TKF via [^11^C]MeOTf were higher than that via ^11^CH_3_I. The biodistribution of [^11^C]-TKF in normal mice revealed that [^11^C]-TKF was metabolized by the gallbladder. Moreover, the brain uptake of [^11^C]-TKF was better than that of [^18^F]-THK523 (3.23% ± 1.25% ID·g^−1^ vs. 2.62% ± 0.39% ID·g^−1^). The acute toxicity of [^11^C]-TKF was negative. Certain parameters can be determined solely by an in vivo administration. For example, the blood-brain barrier penetration is of great importance with regard to the visualization of NFTs in vivo. [^11^C]-TKF displayed excellent brain uptake and could be eluted quickly in normal mice. These findings indicate that [^11^C]-TKF might be a useful PET radiotracer for AD imaging but awaits further evaluation in animal models of AD.

## Figures and Tables

**Table 1:** Comparison of ^11^CH_3_I and [^11^C]MeOTf for radiolabeling of [^11^C]-TKF.

Content	^11^CH_3_I	[^11^C]MeOTf
Precursor	2 mg	1 mg
Total synthesis time (from bombardment)	30–35 min	35–40 min
Reaction time	10 min	3 min
Reaction temperature	140 °C	90 °C
Radiochemical purity	>95%	>95%
yield (decay corrected from radioactivity trapped)	5%–10%	60%–65%
Specific activity	0.4 ± 0.2 Ci/µmol	5.6 ± 0.3 Ci/µmol

**Table 2:** Tissue distribution of radioactivity in mice after injection of [^11^C]-TKF.

Time (s)	% Injection Dose/Tissue ^a^										
	Brain	Heart	Lung	Liver	Gallbladder	Kidney	Stomach	Small Intestine	Muscle	Femur	Blood
20	6.06 ± 1.12	4.24 ± 0.93	3.55 ± 0.71	10.75 ± 0.93	6.18 ± 0.83	2.89 ± 0.82	4.81 ± 0.48	6.16 ± 1.03	2.21 ± 0.66	3.19 ± 0.55	10.05 ± 0.93
40	5.80 ± 1.05	5.23 ± 1.34	3.60 ± 0.66	9.91 ± 2.45	7.63 ± 0.96	2.91 ± 0.61	4.27 ± 1.26	7.99 ± 1.08	1.89 ± 0.62	2.48 ± 0.84	7.24 ± 0.82
60	5.54 ± 1.07	4.67 ± 1.32	3.55 ± 0.31	10.17 ± 0.92	7.07 ± 1.28	2.81 ± 0.44	3.63 ± 0.99	8.58 ± 0.90	2.09 ± 0.46	2.29 ± 0.68	3.38 ± 1.21
90	5.39 ± 0.90	4.63 ± 1.28	2.85 ± 0.34	9.85 ± 2.28	7.32 ± 1.49	3.24 ± 0.85	3.71 ± 0.85	8.12 ± 1.40	1.68 ± 0.55	2.89 ± 0.21	3.16 ± 1.64
120	5.37 ± 1.14	4.59 ± 0.73	3.16 ± 0.99	11.74 ± 1.98	7.91 ± 0.92	2.85 ± 0.44	3.96 ± 0.75	7.71 ± 1.66	1.67 ± 0.61	2.63 ± 1.00	3.70 ± 1.54
180	5.18 ± 1.28	3.95 ± 0.66	2.98 ± 0.78	10.15 ± 1.85	8.86 ± 1.27	2.96 ± 0.42	3.40 ± 0.63	7.15 ± 1.00	1.68 ± 0.57	2.07 ± 0.37	3.64 ± 1.76
240	4.83 ± 1.22	4.42 ± 0.89	2.84 ± 1.03	9.52 ± 1.09	9.68 ± 1.97	2.82 ± 0.20	3.00 ± 0.57	8.86 ± 1.44	1.62 ± 0.58	2.80 ± 0.67	2.99 ± 1.67
300	4.62 ± 1.31	3.58 ± 0.75	3.06 ± 0.47	9.93 ± 1.19	10.60 ± 1.82	2.90 ± 0.64	3.45 ± 0.38	8.81 ± 1.61	1.70 ± 0.39	2.19 ± 0.50	2.71 ± 1.19
450	4.32 ± 1.31	4.01 ± 0.72	2.97 ± 0.72	9.40 ± 0.63	12.77 ± 2.05	2.98 ± 0.29	3.10 ± 0.56	9.15 ± 1.49	1.50 ± 0.25	2.03 ± 0.24	2.22 ± 1.82
600	3.97 ± 1.33	3.83 ± 0.82	2.69 ± 0.73	8.64 ± 1.43	15.00 ± 2.99	3.01 ± 0.48	3.10 ± 0.55	9.64 ± 1.43	1.33 ± 0.35	2.04 ± 0.33	2.66 ± 1.33
750	3.79 ± 1.34	3.67 ± 0.64	2.34 ± 0.33	9.44 ± 1.65	16.71 ± 3.68	3.18 ± 0.32	2.91 ± 0.41	10.95 ± 1.86	1.15 ± 0.32	2.13 ± 0.32	2.39 ± 1.89
900	3.53 ± 1.29	3.54 ± 0.64	2.28 ± 0.57	8.22 ± 0.89	17.66 ± 3.81	2.89 ± 0.51	3.00 ± 0.52	10.81 ± 1.05	1.32 ± 0.31	1.93 ± 0.34	3.17 ± 1.55
1200	3.23 ± 1.25	3.32 ± 0.62	2.51 ± 0.49	8.50 ± 0.33	18.92 ± 4.55	2.68 ± 0.45	2.70 ± 0.70	16.06 ± 2.25	1.00 ± 0.15	1.50 ± 1.17	1.85 ± 1.52
1500	2.88 ± 1.14	3.23 ± 0.39	2.21 ± 0.62	7.86 ± 1.24	20.33 ± 5.25	2.21 ± 0.30	2.61 ± 0.30	18.92 ± 3.06	1.16 ± 0.33	1.64 ± 0.35	1.72 ± 1.59
1800	2.66 ± 1.10	3.29 ± 0.85	2.21 ± 0.40	7.88 ± 0.57	22.03 ± 5.41	1.90 ± 0.49	2.59 ± 0.59	19.82 ± 2.81	1.04 ± 0.34	1.55 ± 0.30	1.65 ± 1.63
2100	2.57 ± 1.08	3.00 ± 0.46	2.04 ± 0.49	7.19 ± 0.34	22.31 ± 5.22	1.88 ± 0.35	2.29 ± 0.28	20.57 ± 3.64	1.16 ± 0.21	1.66 ± 0.32	2.29 ± 1.54
2400	2.33 ± 0.95	2.80 ± 0.62	1.92 ± 0.56	7.27 ± 1.34	23.60 ± 5.11	1.60 ± 0.23	2.49 ± 0.59	20.47 ± 4.07	0.85 ± 0.39	1.57 ± 0.23	1.56 ± 1.76
2700	2.31 ± 1.02	2.97 ± 0.43	1.87 ± 0.21	7.26 ± 1.67	24.62 ± 5.24	1.82 ± 0.41	2.38 ± 0.58	20.49 ± 4.60	0.90 ± 0.42	1.31 ± 0.17	2.33 ± 1.29
3000	2.26 ± 0.98	2.44 ± 0.31	1.80 ± 0.42	6.76 ± 2.34	25.37 ± 4.19	2.34 ± 0.33	2.12 ± 0.78	20.43 ± 3.73	0.96 ± 0.28	1.55 ± 0.18	1.68 ± 1.54
3300	2.22 ± 0.96	3.03 ± 0.42	1.67 ± 0.91	6.80 ± 2.00	27.60 ± 5.20	1.82 ± 0.44	2.30 ± 0.75	21.25 ± 4.29	1.01 ± 0.36	1.13 ± 0.33	1.70 ± 1.31
3600	2.10 ± 0.94	2.51 ± 0.40	1.52 ± 0.23	7.02 ± 1.52	26.55 ± 3.70	2.06 ± 0.48	2.30 ± 0.50	21.52 ± 3.54	1.01 ± 0.62	1.01 ± 0.59	1.57 ± 1.42

^a^ Mean ± S.D. (*n* = 6).
